# MTA1 promotes the invasion and migration of non-small cell lung cancer cells by downregulating miR-125b

**DOI:** 10.1186/1756-9966-32-33

**Published:** 2013-05-29

**Authors:** Yiyi Li, Yilan Chao, Yuan Fang, Jian Wang, Min Wang, Hong Zhang, Min Ying, Xiaoxia Zhu, Haofei Wang

**Affiliations:** 1 Department of Radiation Oncology, Nanfang Hospital, Southern Medical University, Guangzhou, Guangdong 510515, China; 2 Department of Cardiothoracic Surgery, Nanfang Hospital, Southern Medical University, Guangzhou, Guangdong 510515, China

**Keywords:** miR-125b, MTA1, Non-small cell lung cancer, Metastasis

## Abstract

**Background:**

The metastasis-associated gene 1 (MTA1) has been identified as one critical regulator of tumor metastasis. Previously, we identified miR-125b as a downregualted miRNA in non-small cell lung cancer (NSCLC) cell line upon MTA1 depletion. However, the role of miR-125b and MTA1 in the regulation of NSCLC metastasis remains unclear.

**Methods:**

Stable MTA1 knockdown NSCLC cell lines 95D and SPC-A-1 were established by transfection with MTA1 shRNA. The effects of MTA1 depletion on the expression of miR-125b and cell migration and invasion were examined by real-time PCR, wound healing and matrigel invasion assay.

**Results:**

MTA1 knockdown led to the upregulation of miR-125b level in NSCLC cells. Furthermore, MTA1 knockdown reduced while miR-125b inhibitor enhanced cell migration and invasion of NSCLC cells. Notably, miR-125b inhibitor antagonized MTA1 siRNA induced inhibition of cell migration and invasion.

**Conclusion:**

MTA1 and miR-125b have antagonistic effects on the migration and invasion of NSCLC cells. The newly identified MTA1-miR-125b axis will help further elucidate the molecular mechanism of NSCLC progression and suggest that ectopic expression of miR-125b is a potentially new therapeutic regimen against NSCLC metastasis.

## Introduction

Metastasis is the leading cause of failure in clinical treatment of malignant tumors including lung cancer. The metastasis-associated gene 1 (MTA1) has been identified as one critical regulator of the metastasis of many human cancers [1-4]. In our previous studies we deomnstrated that MTA1 promoted the metastasis of non-small cell lung cancer (NSCLC), and identified miR-125b as a downregualted miRNA in NSCLC cell line upon MTA1 depletion [5,6]. However, the role of miR-125b and MTA1 in the regulation of invasive phenotype of NSCLC cells remains unclear.

It has been shown that miR-125b level was significantly correlated with good prognosis of liver cancer [7]. miR-125b was deregulated in lung cancer, oral squamous cell carcinoma, prostate cancer and pancreatic cancer [8-11]. However, controversial properties of miR-125b have been reported in different types of cancer. In human invasive breast cancer, miR-125b functioned as a tumor suppressor by regulating ETS1 proto-oncogene [12]. In addition, miR-125b was underexpressed in metastatic hepatocellular carcinoma (HCC) and inhibited HCC cell migration and invasion by directly targeting oncogene LIN28B2 [13,14]. In contrast, exogenous miR-125b expression increased the migration of type I endometrial carcinoma cell line [15]. Moreover, miR-125b was proposed to function as a metastasis promoter through targeting STARD13 in breast cancer cells [16]. These data suggest that miR-125b may perform different regulatory functions on tumor progression in a cellular context-dependent manner.

In the present study, we established two MTA1-knockdown NSCLC cell lines using stable transfection technology and validated the effects of MTA1 depletion on the expression of miR-125b. Using these cell lines we further examined the function of miR-125b in the regulatuion of cell migration and the interaction between miR-125b and MTA1. Our resutls showed that miR-125b acted as a metastasis suppressor *in vitro* and reversed the stimulatous effect of MTA1 on the migration of NSCLC cell lines.

## Methods

### Cell culture

Human non-small lung cancer cell lines 95D and SPC-A-1 were purchased from Shanghai Cell Bank of Chinese Academy of Science (Shanghai, China). Cells were cultured in RPMI 1640 medium (Invitrogen, Carlsbad, CA) supplemented with 10% fetal calf serum at 37°C in a humidified atmosphere containing 5% CO_2_.

### Transient transfection

miR-125b-inhibitor (5′-UCACAAGUUAGGGUCUCAGGGA-3′) and nonspecific control miRNA (NC, 5′-CAGUACUUUUGUGUAGUACAA-3′) were designed based on miRbase Database (http://www.miRbase.org) and synthesized by Genepharma (Shanghai, China). Cells were seeded (1.6×10^4^/well) onto 96-well plate 18–20 h before transfection. Anti-miR-125b or NC was added to each well. After 6 h incubation at 37°C and 5% CO_2_, the medium was replaced with fresh culture medium. The cells were harvested at 48 h post transfection.

### Establishment of stable cell line

Cells were transfected with 3 μg of plasmids (pLVTHM-MTA1-si, or pLVTHM-CTL-si) which were constructed in previous study [6], or empty pLVTHM vector using Lipofectamine2000 (Invitrogen, Carlsbad, CA) according to the manufacturer’s protocol, then selected for the resistant to neomycin. The stable resistant cell lines were selected and named as 95D (or SPC-A-1)/MTA1-si, 95D (or SPC-A-1)/ CTL-si, and 95D (or SPC-A-1)/NC, respectively.

### Quantitative real-time PCR

Total RNA was extracted from the cells with Trizol reagent (Invitrogen) following the manufacturer’s instruction. Quantitative real-time PCR for miR-125b or MTA1 mRNA was performed as described previously [6]. For miR-125b quantification, U6 small nuclear RNA (U6 snRNA) was used as internal control. The primers sequences were as follows: hsa-miR-125b forward: GGCAACCTTGCGACTATAACCA, reverse: GTTTCCTCTCCCTGAGACCCTA; U6 snRNA forward: CTCGCTTCGGCAGCACATATACT, reverse ACGCTTCACGAATTTGCGTGTC. The relative quantification of expression levels was calculated using the 2^−ΔΔCt^ method.

### Western blot analysis

Total protein was extracted from the cells using RIPA kit (Pierce, USA). Protein concentrations of the supernatants were determined using BCA method. Equal amounts of proteins were separated by SDS-PAGE and transferred into nitrocellulose membranes, which were incubated with primary antibodies against MTA1 (1:1500; Abcam, Cambridge, MA, USA) and β-Actin (1:1000; Santa Cruz Biotech, Santa Cruz, CA, USA) at 4°C overnight. The membranes were washed three times with TBST and incubated with peroxidase conjugated goat anti-rabbit IgG secondary antibody (1:1000, Santa Cruz Biotech, Santa Cruz, CA, USA) for 1 h at room temperature. Finally, the membranes were washed three times with TBST and visualized using Western Blotting Luminol Reagent (Santa Cruz Biotech, Santa Cruz, CA, USA) according to the manufacturer’s instruction.

### Wound healing assay

Cells were seeded into six-well plate and grown to confluence. Wound was created by scraping confluent cell monolayers with a pipette tip. The cells were allowed to migrate for 48 h. At 0 h and 48 h after scratching, images were taken under the inverted microscope to assess the ability of the cells to migrate into the wound area.

### Cell invasion assay

5×10^4^ cells in serum-free media were seeded into the upper chambers of a 24-well BioCoat Matrigel invasion chamber (BD Bioscience, Bedford, MA, USA). Medium with 10% FBS was added to the lower chambers as a chemoattractant. After 24 h of incubation, cells that invaded through the membrane filter were fixed and stained with H&E. The number of invading cells was counted under fluorescence microscope in five random high power fields.

### Statistical analysis

All experiments were repeated independently a minimum of three times, and the results were expressed as the mean values ± standard deviation. The differences between groups were analyzed by two-tailed unpaired Student’s t test. A value of p < 0.05 was considered to indicate statistical significance.

## Results

### MTA1 knockdown leads to the upregulation of miR-125b level in NSCLC cells

**Figure 1:**
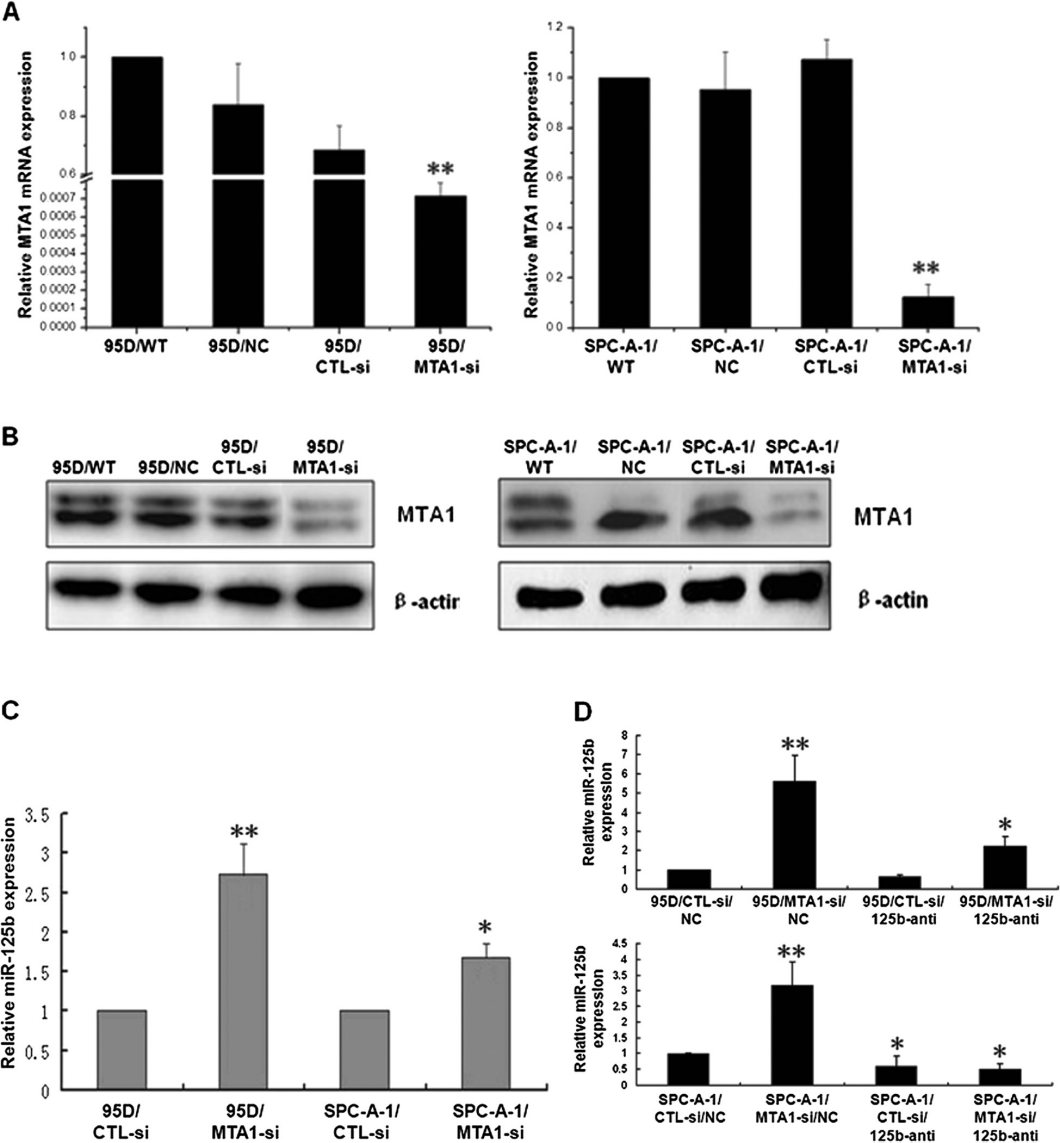
**MTA1 knockdown leads to the upregulation of miR-125b level in NSCLC cells. A**. Quantification of MTA1 mRNA level by quantitative RT-PCR in 95D and SPC-A-1 cells untransfected, transfected with MTA1 shRNA or control shRNA. **B**. Western blot analysis of MTA1 protein level in 95D and SPC-A-1 cells untransfected, transfected with MTA1 shRNA or control shRNA. B-actin was loading control. **C**. Quantification of miR-125b level by quantitative RT-PCR in 95D and SPC-A-1 cells transfected with MTA1 shRNA or control shRNA. **D**. Quantification of miR-125b level by quantitative RT-PCR in 95D and SPC-A-1 cells transfected with MTA1 shRNA or control shRNA, together with miR-125b inhibitor or control. *P < 0.05, **P < 0.01 compared to the controls.

First we established 95D and SPC-A-1 cell lines with stable knockdown of MTA1 by transfecting the cells with MTA1 shRNA. The knockdown efficiency was confirmed by qRT-PCR and Western blot analysis. Compared to the control cell lines, the expression of MTA1 mRNA and protein was significantly reduced in 95D and SPC-A-1 cells transfected with pLVTHM-MTA1-si plasmid (Figure 1A, B).

Next we detected miR-125b level in the established cell lines. The results showed that miR-125b level was 2.75 and 1.67-fold higher in 95D/MTA1-si and SPC-A-1/MTA1-si cells, compared to the control 95D and SPC-A-1 cells, respectively (Figure 1C). To confirm the negative correlation between MTA1 and miR-125b in NSCLC cells, we transfected miR-125b-inhibitor or nonspecific control miRNA (NC) into 95D and SPC-A-1 cells. qRT-PCR analysis showed that miR-125b-inhibitor decreased the expression of miR-125b in 95D/CTL-si and SPC-A-1/CTL-si cells only by 30 percent, but it significantly reduced miR-125b expression in 95D/MTA1-si and SPC-A-1/MTA1-si cells (Figure 1D). These data suggest that MTA1 knockdown leads to the upregulation of miR-125b level in NSCLC cells.

### MTA1 and miR-125b have antagonistic effects on the migration and invasion of NSCLC cells

**Figure 2:**
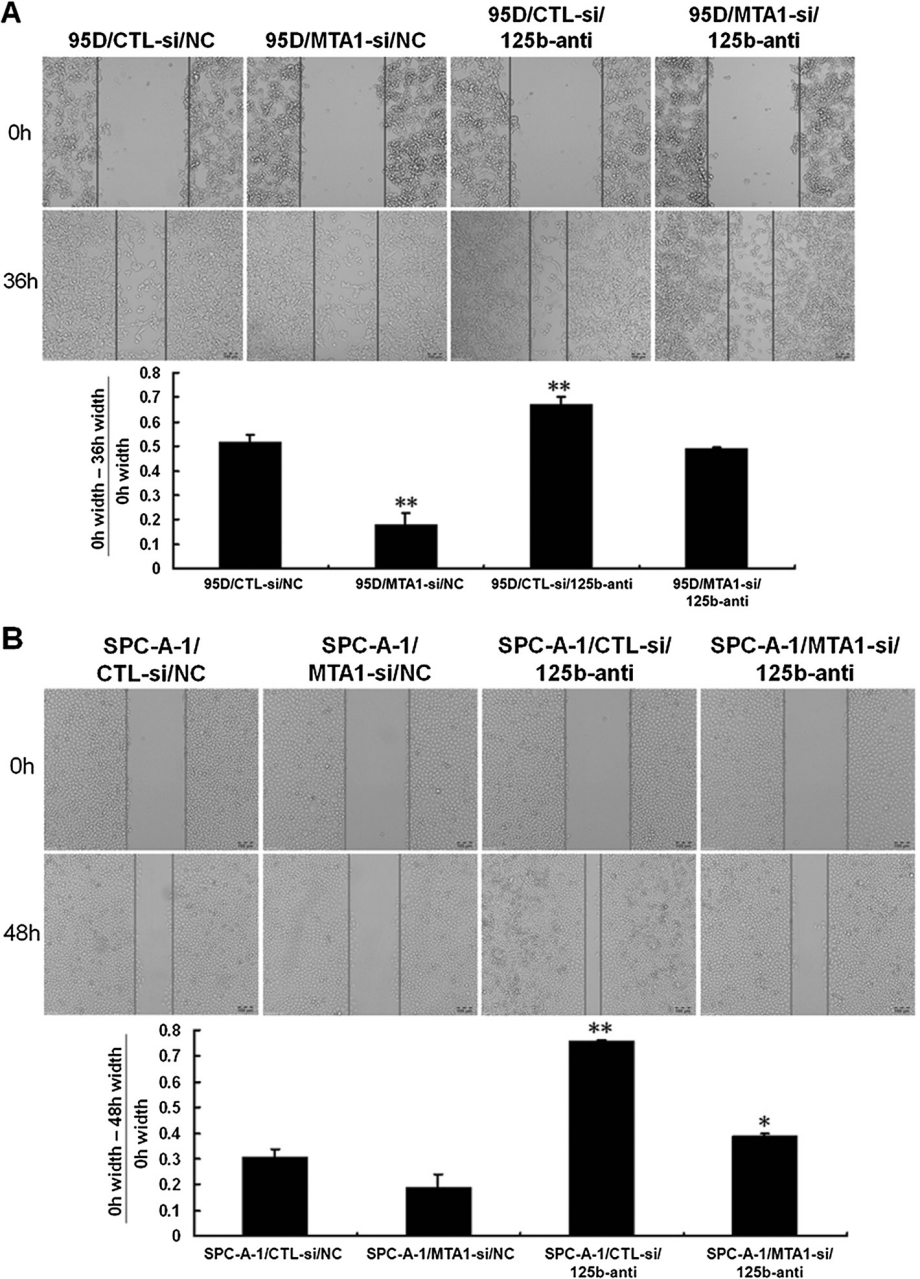
**MTA1 and miR-125b have antagonistic effects on the migration of NSCLC cells. A**. Wound healing assay on the migration of 95D cells transfected with MTA1 shRNA or control shRNA, together with miR-125b inhibitor or control. The percentage of the wound healing was calculated as (the width of wound at 0 h - the width of wound at 36 h)/ the width of wound at 0 h. **P < 0.01 compared to controls. **B**. Wound healing assay on the migration of SPC-A-1 cells transfected with MTA1 shRNA or control shRNA, together with miR-125b inhibitor or control. The percentage of the wound healing was calculated as (the width of wound at 0 h - the width of wound at 48 h)/ the width of wound at 0 h. *P < 0.05, **P < 0.01 compared to controls.

Next we investigated the antagonistic effects of MTA1 and MiR-125b on the migration and invasion of NSCLC cells. Wound healing assay showed that in 95D cells, knockdown of MTA1 led to reduced cell migration. However, cell migration was increased in 95D cells treated with miR-125b inhibitor. Moreover, the migration of cells treated with both MTA1 shRNA and miR-125b inhibitor was similar to control cells (Figure 2A). Similar results were observed for the migration of SPC-A-1 cells (Figure 2B). These data demonstrate that MTA1 promotes while miR-125b inhibits NSCLC cell migration and indicate that MTA1 may promote cell migration via the downregulation of miR-125b.

**Figure 3:**
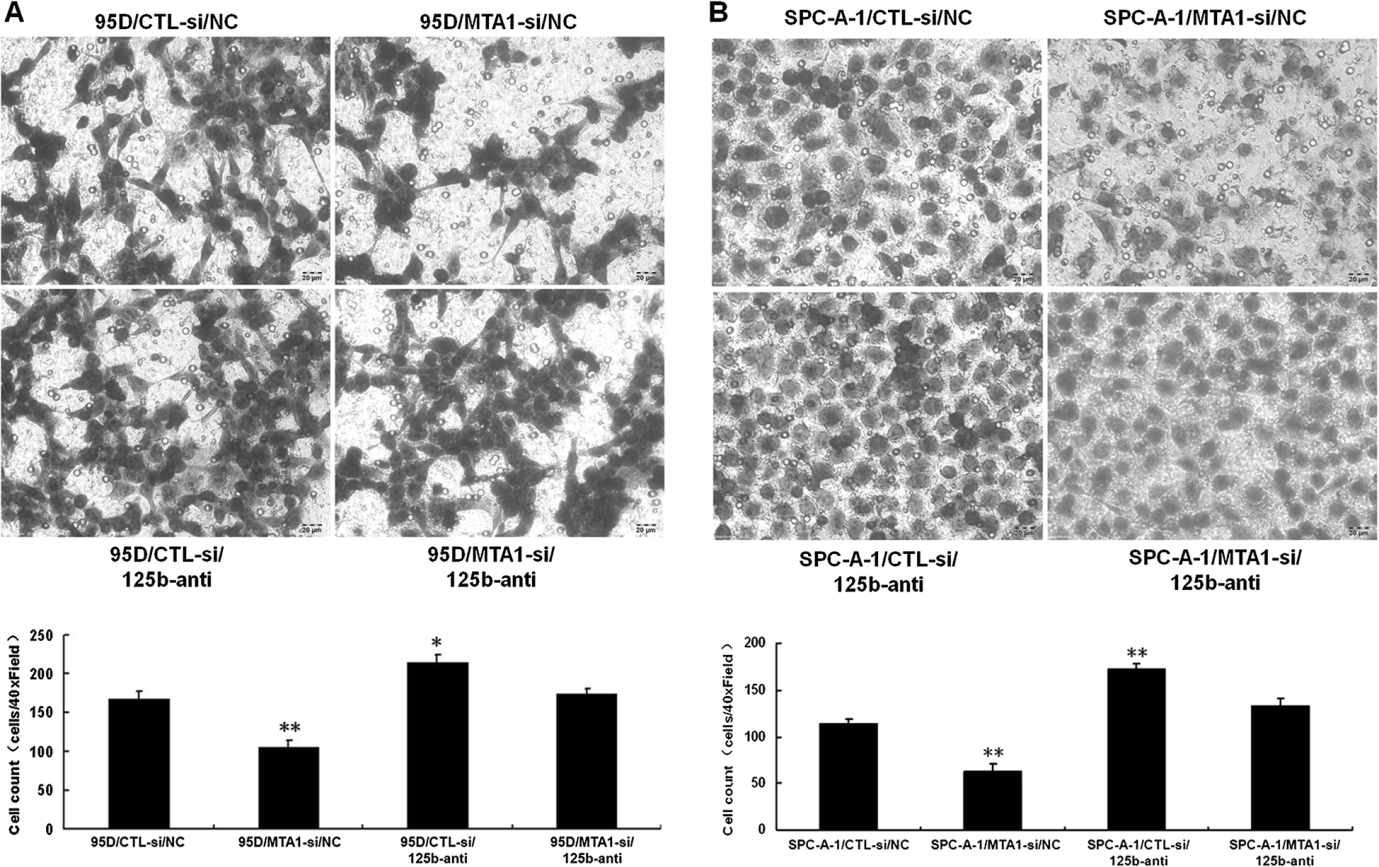
**MTA1 and miR-125b have antagonistic effects on the invasion of NSCLC cells. A**. Transwell invasion assay on the invasion of 95D cells transfected with MTA1 shRNA or control shRNA, together with miR-125b inhibitor or control. The invaded cells were counted from 5 random fields at 40x magnification. *P < 0.05, **P < 0.01 compared to controls. **B**. Transwell invasion assay on the invasion of SPC-A-1 cells transfected with MTA1 shRNA or control shRNA, together with miR-125b inhibitor or control. The invaded cells were counted from 5 random fields at 40x magnification. **P < 0.01 compared to controls.

Matrigel invasion assay showed that in 95D cells, knockdown of MTA1 led to reduced cell invasion. However, cell invasion was increased in 95D cells treated with miR-125b inhibitor. Moreover, the invasion of cells treated with both MTA1 shRNA and miR-125b inhibitor was similar to control cells (Figure 3A). Similar results were observed for the invasion of SPC-A-1 cells (Figure 3B). These data demonstrate that MTA1 promotes while miR-125b inhibits NSCLC cell invasion and indicate that MTA1 may promote cell invasion via the downregulation of miR-125b.

## Discussion

Recent studies have demonstrated the crucial role of miR-125b in tumorigenesis and metastasis [17-20]. Nevertheless, the role of miR-125b in lung cancer remains controversial. chr11q23-24 and chr21q11-21 are the region in which miR-125b-1 and miR-125b-2 are located, respectively, and they are frequently deleted in patients with lung cancer, indicating that miR-125b may function as a tumor suppressor in lung cancer [8,21]. However, miR-125b exhibited higher expression level in non-responsive patients with cisplatin-based chemotherapy [22]. Furthermore, the high level of miR-125b was significantly correlated with poor patient survival [22,23]. These data suggest that miR-125b may act as an oncogene in lung cancer.

In the present study, by cell biological analysis we demonstrated that inhibition of miR-125b promoted the migration and invasion of NSCLC cells, providing some evidence that miR-125b could serve as a tumor suppressor in the metastasis of NSCLC *in vitro*. The upstream regulators of miR-125b expression remain to be identified. Recently Liu *et al.* reported that STAT3 could promote the transcription of miR-125b in human osteosarcoma cells [24]. In addition, CDX2, a homeobox transcription factor, has been recently shown to bind to the promoter region of miR-125b and activate its transcription in malignant myeloid cells [25]. By microarray analysis, we previously found that miR-125b was significantly upregulated in MTA1 knockdown NSCLC cells [6]. In this study, we verified that endogenous expression of miR-125b increased after the depletion of MTA1 in two NSCLC cell lines, suggesting that miR-125b is regulated by MTA1 at the level of transcription. Furthermore, we found that the inhibition of miR-125b could rescue the suppressive effects of MTA1 silencing on NSCLC cell migration and invasion. These results demonstrate for the first time that miR-125b is a functional target of MTA1 in lung cancer cells and suggest that ectopic expression of miR-125b is a promising strategy to counteract the promotion of tumor progression by MTA1.

It is known that MTA1, which is an integral part of nucleosome remodeling and deacetylation (NuRD) complexes, represses the transcription of target genes by recruiting histone deacetylases onto the promoter regions of target genes and inducing histone deacetylation [25]. Further studies are needed to elucidate the mechanism by which MTA1 downregulates the transcription of miR-125b in lung cancer cells.

## Conclusions

In summary, we found that the expression of MTA1 and miR-125b is negatively correlated in lung cancer cells and they have antagonistic effects on the migration and invasion of NSCLC cells. The newly identified MTA1-miR-125b axis will help further elucidate the molecular mechanism of NSCLC progression and suggest that ectopic expression of miR-125b is a potentially new therapeutic regimen against NSCLC metastasis.

## Competing interests

The authors declare that they have no competing interests.

## Authors’ contributions

YL, YC, YF, JW, MW performed most of the experiments. HW and XZ designed the study. HZ and MY performed statistical analysis. XZ supervised the study and wrote the manuscript. All authors read and approved the final manuscript.
